# Patient Perspectives on Value Dimensions of Lung Cancer Care: Cross-sectional Web-Based Survey

**DOI:** 10.2196/37190

**Published:** 2023-01-26

**Authors:** Pasquale Varriale, Borna Müller, Grégory Katz, Lorraine Dallas, Alfonso Aguaron, Marion Azoulai, Nicolas Girard

**Affiliations:** 1 Carenity part of EvidentIQ group Paris France; 2 Global Access F Hoffmann-La Roche Ltd Basel Switzerland; 3 Université Paris-Cité Medical School Paris France; 4 PromTime Paris France; 5 Roy Castle Lung Cancer Foundation London United Kingdom; 6 Lung Cancer Europe Madrid Spain; 7 Institut du Thorax Montsouris, Institut Curie Paris France; 8 Paris Saclay University Université Versailles Saint Quentin Paris France

**Keywords:** lung, cancer, health quality of life, patient reported outcome, PROM, economic burden, cost, economic, burden, perspective, survey, QoL, quality of life, questionnaire, caregiver, caregiving, physical well-being, end of life, palliative, physical function, independence, distress

## Abstract

**Background:**

While the lung cancer (LC) treatment landscape has rapidly evolved in recent years, easing symptom burden and treatment side effects remain central considerations in disease control.

**Objective:**

The aim of this study was to assess the relative importance of dimensions of LC care to patients, and to explore the disease burden, including socioeconomic aspects not commonly covered in patient-reported outcomes instruments.

**Methods:**

A questionnaire was sent to patients with LC and their caregivers to rate the value of a diverse set of quality of life dimensions in care, to evaluate communication between health care professionals (HCPs) and patients, and to explore the economic impact on respondents. The survey included questions on the dimensions of care covered by patient-reported outcomes instruments for quality-of-life evaluation (Functional Assessment of Cancer Therapy–Lung scale, EQ-5D, the European Organization for Research and Treatment of Cancer’s Core Quality of Life questionnaire, and the European Organization for Research and Treatment of Cancer’s Core Quality of Life in lung cancer 13-item questionnaire), as well as the International Consortium for Health Outcomes Measurement (ICHOM) standard set of patient-centered outcomes for LC. The survey respondents were participants on Carenity’s patient community platform, living either in France, the United Kingdom, Germany, Italy, or Spain.

**Results:**

The survey included 150 respondents (115 patients and 35 caregivers). “Physical well-being” and “end-of-life care” (median scores of 9.6, IQR 7.7-10, and 9.7, IQR 8.0-10, on a 10-point scale) were rated highest among the different value dimensions assessed. “Physical well-being and functioning” was the dimension most frequently discussed with health care professionals (82/150, 55%), while only (17/100, 17%) reported discussing “end-of-life care.” After diagnosis, 43% (49/112) of patients younger than 65 years stopped working. Among respondents who indicated their monthly household income before and after diagnosis, 55% (38/69) reported a loss of income.

**Conclusions:**

Our results showed the relevance of a broad range of aspects of care for the quality of life of patients with LC. End-of-life care was the dimension of care rated highest by patients with LC, irrespective of stage at diagnosis; however, this aspect is least frequently discussed with HCPs. The results also highlight the considerable socioeconomic impact of the disease, despite insurance coverage of direct costs.

## Introduction

In 2018, over 2 million new lung cancer (LC) cases were diagnosed worldwide, accounting for 19% of all cancer-related deaths in men and women [1]. Often diagnosed at a late stage, more than half of patients with LC die within the first year after diagnosis, with a 5-year survival rate of less than 20% [2,3].

The LC treatment landscape is rapidly evolving, with many new therapeutic options and an increasing focus on personalized approaches [2]. In addition to using cytotoxic chemotherapy, LC management today also considers staging and patients’ clinical characteristics, with the identification of oncogenic driver alterations and other predictive factors. There is increasing use of targeted therapies, immune checkpoint inhibitors, and chemo-immunotherapy. Nevertheless, though efficacy and tolerability of treatments have improved significantly in recent years, patients remain clinically vulnerable, and symptom burden and treatment side effects remain key considerations in disease control [2,4].

In this context, measuring treatment outcomes from the patient’s perspective is critical to assessing the value of an episode of care in terms of functional recovery and quality of life (QoL) in daily activities.

Patient-reported outcome measures (PROMs) use psychometric instruments such as calibrated auto-questionnaires to quantify patient health gains, especially health-related quality of life (HRQOL). HRQOL is a multidimensional outcome measuring patients’ physical, psychological, and social status over a care pathway. It is universally considered a measure of clinical benefit, ensuring a patient-centered treatment approach. There are a number of HRQOL measurement tools currently used for patients with LC in both clinical trials and daily clinical practice [4-10].

Using PROMs in the context of LC management is beneficial in several respects. PROMs can provide a more complete assessment of the benefits and risks of treatment by helping to monitor the response and identify adverse events to therapy. Moreover, when used in daily practice, PROMs may support patient–health care professional (HCP) communication [7,11,12]. Furthermore, growing evidence supports the idea that QoL measures can provide an independent prognostic factor for patients with LC [6,13,14].

Consequently, scientific societies in oncology and regulatory agencies in the United States and Europe have underlined the importance of QoL measurements. They highlight the relevance of the patient’s perspective as a standard outcome measure and the importance of incorporating PROMs in the evaluation of treatment in both clinical trials and clinical practice [9,15,16]. Nevertheless, despite this accepted importance and increasing use, PROMs are still insufficiently incorporated into clinical trials and clinical practice [7,12,15,17-32].

Combined with other data sources such as clinician-reported outcome measures or cost analysis, PROMs must be considered as part of a broader toolkit for assessing the impact of LC on patients, caregivers, and health systems [19,33-35].

Besides the importance of using PROMs associated with care, there is a need for consensus on what health domains to assess in cancer with this tool [36]. Moreover, assessments of extraclinical outcomes are not typically incorporated in PROMS to assess impact on QoL. However, a working group from the International Consortium for Health Outcomes Measurement (ICHOM) has published a standard set of patient-centered outcomes for LC, including value dimensions not covered by most PROMs commonly in use [37].

The aim of this study was to assess the dimensions of LC care most relevant to patients. Furthermore, we explored the burden of LC on patients and caregivers (including economic aspects not commonly covered in PROMs), the importance of care management, current perspectives on medical priorities, and the differences in value perception among patients, caregivers, payers, and providers by means of a cross-sectional web-based survey on the Carenity patient community platform.

## Methods

### Study Design and Population

Data collection was conducted by Carenity, a web-based patient community with more than 400,000 patients and caregivers covering 1200 chronic conditions across Europe and the United States [38]. Patient recruitment on Carenity is mainly done digitally, using both free and paid methods (Multimedia Appendix 1).

The survey was conducted from August 12, 2019, to November 8, 2019. All adult patients or caregivers of patients registered with LC living in France, the United Kingdom, Germany, Italy, or Spain were invited, via emails and private messages on the Carenity platform, to participate in a confidential web-based satisfaction survey. Thus, the study population consisted of a voluntary sample of patients and caregivers who completed the survey. To exclude double counting of responses from cases where patients, as well as relatives and caregivers, simultaneously responded to the survey, the patient’s sociodemographic profiles indicated by the respondents were systematically compared. Patients did not receive any compensation for this survey, which was entirely voluntary.

### Survey Questionnaire

A specific questionnaire was designed to measure the aspects of QoL considered most important by patients and their caregivers. This questionnaire covered all dimensions covered by the most commonly used PRO (patient-reported outcomes) instruments for QoL evaluation of patients with LC [7] (Functional Assessment of Cancer Therapy–Lung scale, EQ-5D, the European Organization for Research and Treatment of Cancer’s Core Quality of Life questionnaire, and the European Organization for Research and Treatment of Cancer’s Core Quality of Life in lung cancer 13-item questionnaire), as well as the ICHOM standard set of patient-centered outcomes for LC.

Elements that could be relevant for patients but were not covered in the PRO instruments or the ICHOM standard set, such as the economic burden of the disease, were also added.

The study questionnaire included 6 QoL dimensions: “physical functioning and well-being,” “emotional well-being,” “daily life,” “medical care,” “treatment,” and “end-of-life.” Each dimension included several subdimensions. Main dimensions, subdimensions, and the tools from which they were derived are provided in Textbox 1.

The importance of each dimension and subdimension for the patients or their caregivers was assessed using a numeric scale, with 0 indicating the lowest relevance to patients and 10 the highest.

In addition, respondents were asked whether it was important for them to have these dimensions evaluated by their physician. They were also asked which dimensions had been discussed with their physicians in their interactions.

Caregivers were mainly asked to respond from the patient’s perspective. Specific questions regarding their sociodemographic information or relationship to the patients were also included. Caregiver’s value of the “daily life” dimension (with subdimensions of emotional well-being, physical well-being, family life, romantic relationships, professional life, and purchasing power) and evaluation by HCPs of the impact of LC on caregivers’ daily lives were also assessed, with the question being asked either directly to them or to the patient (answering from the caregiver’s point of view). A full version of the questionnaire can be found in Multimedia Appendix 2.

The protocol was revised by a board of experts to ensure that the burden on patients was assessed and that the time spent answering all the questions was not longer than 15 minutes.

**Definition of quality-of-life dimensions and subdimensions considered in the study.**

**Physical functioning and well-being**

- Physical well-being
- Autonomy
- Mobility

EQ-5D-5L/Functional Assessment of Cancer Therapy–Lung (FACT-L) questionnaire/the European Organization for Research and Treatment of Cancer’s Core Quality of Life questionnaire (QLQ-C30) + Quality of Life in lung cancer 13-item questionnaire (QLQ-LC13)

**Emotional well-being**

- Emotional well-being
- Emotional support from family and friends
- Self-acceptance
- Not being judged or blamed by others

EQ-5D-5L/FACT-L questionnaire/QLQ-C30 + QLQ-LC13

**Daily life**

- Social life
- Family life
- Romantic relationship
- Sexual life
- Leisure purchasing power or standard of living

EQ-5D-5L/FACT-L questionnaire/QLQ-C30 + QLQ-LC13

**Medical care**

- Easy access to place of care
- Less overnight time spent at place of care
- Low frequency of medical follow-up
- Relationship with health care professionals

EQ-5D-5L/FACT-L questionnaire/QLQ-C30 + QLQ-LC13

**Treatment**

- Possibility to take the treatment by myself or by themselves
- Convenience of the route of administration
- Treatment side effects
- Logistics to get the treatment
- Professional life

International Consortium for Health Outcomes Measurement (ICHOM) Standard Set

**End-of-life**

- Place of death
- Presence of loved ones at moment of death
- Pain management
- End-of life assistance
- Duration of end-of-life hospitalization
- Involvement in end-of-life care decisions or respect of living will
- Financial impact on loved ones

ICHOM Standard Set

### Statistical Analysis

Patients’ sociodemographic and clinical characteristics were described using summary statistics. Continuous variables were reported as median with IQR or mean with SD values. Categorical variables were reported as counts and proportions. The number and percentage of patients with missing data for each variable were described.

The scores evaluating the importance of each dimension were described using mean and median. Statistical analysis was performed in R (version 3.6.1; R Core Team), and for all tests, statistical significance was assumed at *P*<.05. Differences in responses to categorical measures were tested using the chi-square test. For continuous variables, a 1-way ANOVA was used.

### Ethics Approval

Respondents participating in the study provided informed consent to the collection, handling, and storage of their personal and health data. It was conducted on the internet, and no HCPs were involved in the patient’s recruitment. The study fell under and complied with national regulations for market research or “patient satisfaction surveys” and followed best practice guidelines [39,40]. No assessment by an ethics committee was required in any of the countries involved. Specifically, we observed the following national guidelines to evaluate the requirement for an ethics assessment: France: Public Health Code, Title II: Research involving humans (Article L1121-1), 2nd point [41]; Germany: Joint recommendations of Bundesinstitut für Arzneimittel und Medizinprodukte and Paul-Ehrlich-Institut on observational studies: §67, section 6 of the Medicinal Product Act [42]; Spain: Farmaindustria–code of practice [43]; United Kingdom: Health Research Authority Defining Research Table [44]; Italy: classification of observational studies requiring ethical assessment by the Italian medicine agency from March 20, 2008 [45]

## Results

### Respondent Profile

The survey included 150 respondents out of the 212 who started it. The completion rate across all countries was 70% (150/214). Among the 150 respondents, 115 (77%) were patients and 35 (33%) were caregivers (responding on behalf of patients). The 150 respondents were similarly distributed between participating countries, from 22 (15%) in Germany to 37 (25%) in France. Sociodemographic and clinical characteristics of the patients are provided in Table 1. The sociodemographic characteristics of the caregivers are also presented (Multimedia Appendix 3).

Men represented 45% (68/150) of patients. The mean age was 58.8 years, with 23% (34/150) being older than 65 years; 21% (32/150) of patients were current smokers, and 65% (97/150) were former smokers (31%, 47/150 had quit after diagnosis, 34% 50/150 before diagnosis).

Most of the patients (90/150, 61%) were diagnosed with LC less than 1 year before inclusion. According to the participants’ responses, 54% (81/150) of the patients had localized cancer, 25% (38/150) had advanced cancer, 3% (5/150) were in remission, and 17% (26/150) did not know the stage of their disease. With regards to treatment, 27% (40/150) stated that they were in remission, 32% (49/150) were under curative care, 11% (16/150) were under palliative care, and 30% (45/150) did not respond to this question.

Caregivers were mostly women (33/35, 94%) and either a relative of a patient (32/35, 91%), such as their child (16/35, 45%), or their partner (14/35, 40%). They were mostly younger than 56 years (29/35, 83%). Overall, they represented between 9% (2/22; Germany) and 41% (15/37; France) of respondents.

**Table 1:** Patients’ sociodemographic and medical characteristics (N=150).

Characteristics		Patients, n (%)
**Geographic segmentation, n (%)**		
	France	37 (25)
	Spain	33 (22)
	Italy	30 (20)
	United Kingdom	28 (19)
	Germany	22(14)
**Male gender, n (%)**		68 (45)
Age (years), mean (SD)		58.8 (9.6)
Age groups (years), n (%)		
	<46	11 (7)
	46-55	35 (25)
	56-65	67 (45)
	>65	34 (23)
**Smoking status, n (%)**		
	Current smokers	32 (21)
	Former smoker (quit after diagnosis)	50 (34)
	Former smoker (quit before diagnosis)	47 (31)
	Never smoker	21 (14)
**Time since diagnosis (years), n (%)**		
	<1	90 (61)
	≥1	60 (39)
**Lung cancer stage, n (%)**		
	Localized	81 (54)
	Advanced	38 (25)
	In remission	5 (3)
	I don’t know or I do not want to answer	26 (17)
**Lung cancer treatment status, n (%)**		
	Under curative treatment	49 (32)
	Under palliative treatment	16 (11)
	In remission	40 (27)
	I don’t know or I do not want to answer	45 (30)

### Prioritization of QoL Dimensions and Medical Assessment to Patients With LC and Caregivers

#### Importance of QoL Dimensions and HCP Assessment to Patients

All QoL dimensions defined in the study questionnaire received high scores from survey participants. “End-of-life care” was rated the most important main dimension (median score 9.7 (IQR 8.0-10) on a 10-point scale; Table 2). The subdimensions of “end-of-life care” also received very high scores and constituted 5 out of the 10 most highly scored subdimensions (Textbox 1). “Pain management,” “presence of loved ones at time of death,” “involvement of caregivers in decision,” “duration of hospitalization,” and “place of death” had a median score equal to or greater than 9.4 on a 10-point scale (Table 2). Among patients who reported the stage of their disease (119 out of 150 patients), these subdimensions remained highly valued by both patients with localized as well as advanced stages of LC (range for patients with localized disease: 8.7 to 9.8; for patients with advanced disease, scores were 10 for all subdimensions; data not shown).

“Physical well-being” was the second most important main dimension (with a median score of 9.6, IQR 7.7-10, on a 10-point scale), while the “daily life” dimension had the lowest score (a median score of 6.9, IQR 3.1-9.5). All other dimensions had a median score of more than 8.6 (Table 2).

**Table 2:** Scores for quality-of-life dimensions and subdimensions for all respondents and stratified by men and women (*P* values indicate level of significance for differences between men and women).

Dimension and subdimension		All (n=150), median (IQR)	Men (n=68), median (IQR)	Women (n=82), median (IQR)	*P* value
**Physical functioning and well-being**					
	Physical well-being	9.4 (7.7-10.0)	8.9 (7.2-10.0)	9.8 (7.9-10)	—^a^
	Autonomy	9.8 (7.6-10.0)	9.1 (6.6-10)	9.9 (8.5-10.0)	.03
	Mobility	9.6 (8.0-10.0)	9.2 (7.6-10)	9.6 (8.5-10.0)	—
**Emotional well-being**					
	Emotional well-being	9.3 (7.4-10.0)	8.6 (6.4-10)	9.8 (8.2-10.0)	.02
	Emotional support	9.5 (7.6-10.0)	9.2 (7.0-10.0)	9.7 (8.4-10.0)	.02
	Self-acceptance	8.5 (5.9-9.9)	7.5 (4.7-9.4)	9 (7.4-10.0)	.001
	No judgment	6.6 (2.1-9.5)	5.9 (1.8-9.3)	7.2 (3.1-9.6)	—
**Daily life**					
	Social life	6.9 (4.9-8.7)	6.3 (3.4-8.6)	7 (5.3-8.7)	—
	Family life	8.8 (7.0-10.0)	8.5 (6.5-10.0)	9 (7.5-10.0)	—
	Romantic relationship	6.9 (1.6-9.8)	7.4 (2.1-9.9)	6 (1.2-9.8)	—
	Sexual life	4.2 (1.0-8.0)	4.5 (1.1-8.6)	3.2 (0.9-6.7)	—
	Leisure	6.6 (4.5-8.7)	5.9 (3.5-8.5)	7 (4.9-8.8)	—
	Purchasing power	7.5 (5.3-9.9)	7.2 (4.6-9.9)	7.7 (5.4-10.0)	—
	Professional life	4.5 (0.9-8.3)	4.2 (0.8-8.0)	4.6 (1.1-8.9)	—
**Medical care**					
	Easy access to place of care	9.1 (7.1-10.0)	8.7 (6.7-9.9)	9.5 (7.4-10.0)	—
	Less overnight time spent at place of care	8.4 (5.2-10.0)	7.3 (4.3-9.4)	8.9 (6.3-10.0)	.01
	Low frequency of medical follow-up	8.5 (5.7-10.0)	7.7 (4.8-9.8)	9 (7.3-10.0)	.004
	Relationship with health care professionals	9.8 (8.2-10.0)	9.3 (7.5-10.0)	9.9 (8.5-10.0)	.04
**Treatment**					
	Take treatment by myself	8.4 (4.8-10.0)	8 (4.7-9.9)	8.5 (4.9-10.0)	—
	Convenience of administration	8.7 (6.6-10.0)	8.1 (5.6-9.7)	9.3 (7.5-10.0)	.007
	Side effects	9.2 (7.7-10.0)	8.9 (7.4-10.0)	9.7 (7.9-10.0)	—
	Logistics to get treatment	8.7 (6.3-10.0)	8.6 (5.8-10.0)	8.7 (6.4-10.0)	—
**End-of-life (n=100, M=46, W=54)**					
	Place of death	9.4 (6.4-10.0)	9.2 (5.8-10.0)	9.6 (7.3-10.0)	—
	Presence of loved ones	9.8 (8.0-10.0)	9.3 (5.2-10.0)	10 (9.1-10.0)	.006
	Pain management	10.0 (9.2-10.0)	9.8 (9.2-10.0)	9.9 (9.3-10.0)	—
	End-of-life support	9.1 (6.7-10.0)	8.4 (6.0-10.0)	10 (8.0-10.0)	.04
	Duration of hospitalization	9.5 (7.8-10.0)	8.8 (5.1-9.9)	10 (8.4-10.0)	.007
	Involvement caregiver in decisions	9.7 (7.8-10.0)	8.8 (6.0-10.0)	10 (8.4-10.0)	.01
	Financial impact on loved ones	9.4 (5.6-10.0)	9.1 (5.6-9.3)	9.8 (6.6-10.0)	—

^a^Not determined.

In addition to rating the overall importance of various QoL dimensions, patients were also asked to indicate the relevance of discussing them with their HCP. Discussing “physical functioning and well-being” was reported as most important (114/150, 76% of patients reported that it was important to discuss with their HCP), while “end-of-life care” scored lower (65/150, 43%). However, in both cases, a lower proportion of patients reported actually having discussed “physical functioning and well-being” and “end-of-life care” with their HCPs (83/150, 55% and 36/150, 17%, respectively, confirmed having discussed this). Among all QoL dimensions assessed, “end-of-life care” was discussed with an HCP the least often.

**Figure 1:**
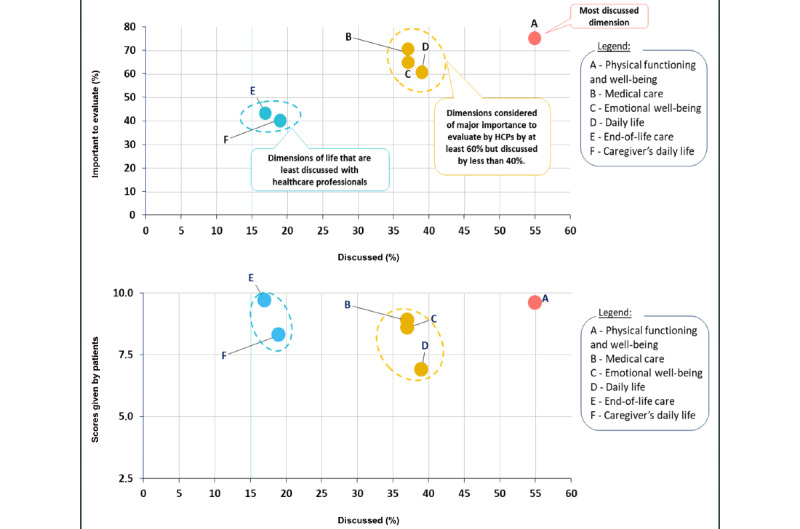
QoL dimensions evaluated by HCP. Upper part: The x-axis indicates the proportion of patients stating that the respective dimension was evaluated by their HCP; the y-axis indicates the proportion of patients indicating these dimensions to be important for discussions with their HCP. Lower part: The x-axis indicates the proportion of patients who state that the respective dimension was evaluated by their HCP; the y-axis shows the average value score given by patients. HCP: health care professional; QoL: quality of life.

Another dimension that scored highly was “medical care” (median score of 8.9, IQR 6.7-10). Its subdimension “relationship with health care professionals” obtained the highest score among all subdimensions in this category (median 9.8, IQR 8.2-10; Table 2). The 3 remaining subdimensions of “medical care” were also considered very important: “easy access to place of care,” “low frequency of medical follow up,” and “less overnight time spent at place of care.” Most patients (103/150, 69%) also considered it important to discuss “medical care” with HCPs, but only a minority (38/103, 37%) reported actually having discussed it with them (Figure 1).

A discrepancy between the overall importance of a dimension for patients to be evaluated by HCPs and the number of patients who reported discussing that dimension with their HCP was reflected in other dimensions as well. For example, both “emotional well-being” (98/150, 65%) and “daily life” (92/150, 61%) scored highly, even though less than 40% (39/98) and 37% (34/92), respectively, reported discussing them with HCPs (Figure 1).

#### Caregiver-Relevant Importance of “Daily Life” Dimensions and HCP Assessment

We also assessed the importance of specific aspects of daily life for caregivers of patients with LC. To do this, we asked caregivers to rate the importance of subdimensions of daily life for themselves (n=35) or patients to provide ratings on behalf of their caregivers (n=115).

Except for professional life, both groups reported similar, consistent scores, supporting the validity of the answers to evaluate the relevance of these subdimensions of daily life for caregivers. The data showed that “physical well-being,” “emotional well-being,” and “family life” were relatively more relevant to caregivers than “romantic relationship,” “purchasing power,” and “professional life.” However, the latter still shows high ratings (Table 3). The significantly different scoring for “professional life” based on whether the respondent was a caregiver or a patient may suggest a different interpretation of the question and consequently allows limited interpretation. Comparing the relevance of these dimensions for caregivers with the ratings given for patients with LC (Multimedia Appendix 4) shows a similar ranking of value dimensions for patients as well as caregivers (data not shown).

Respondents also reported that HCPs rarely assessed the burden of a patient’s LC illness on their caregivers. Overall, less than 20% (28/150) of the respondents indicated that this had been discussed with their HCP (Figure 1).

**Table 3:** Median scores for “daily life” subdimensions as reported by caregivers from their own perspective (N=35) and patients on behalf of their caregivers (N=115; see question 26 from Multimedia Appendix 2).

Dimension and subdimension	Patients (n=115), median (IQR)	Caregivers (n=35), median (IQR)	*P* value
Physical functioning and well-being	9.4 (6.2-10.0)	8.7 (6.7-10.0)	—^a^
Emotional well-being	9.3 (6.4-10.0)	8.8 (6.8-10.0)	—
Family life	8.8 (6.2-10.0)	8.9 (6.0-10.0)	—
Romantic relationship	6.9 (3.5-9.6)	7.4 (3.8-9.6)	—
Professional life	4.5 (2.2-7.0)	7.2 (3.3-9.6)	.005
Purchasing power	7.5 (4.8-10.0)	7.7 (4.9-9.8)	—

^a^Not determined.

### Gender and Country Subgroup Analysis

In general, women scored all QoL aspects higher than men. These differences were particularly pronounced in some areas (Table 2). For example, in 3 out of the 4 subdimensions of the 2 dimensions, “emotional well-being” and “medical care,” women and men scored significantly differently. For “end-of-life,” the scores for 5 of the 7 subdimensions also showed substantial differences. For “physical well-being and functioning,” women scored significantly higher than men on the “autonomy” subdimension. In “treatment,” women also scored the “convenience of administration” subdimension higher than men. Only the “romantic relationship” and “sexual life” subdimensions were rated higher by men than women (respective medians of 7.4 vs 6.0 for women and 4.5 vs 3.2 for women without significant differences; Table 2).

We also observed differences in the scores attributed to the different QoL dimensions and subdimensions between countries. It appeared that UK patients were less inclined to answer questions about end-of-life than patients from other countries, especially Germany (57% (16/28) in the United Kingdom vs 77% (17/22) in Germany). Most importantly, only 32% (12/37) of the French and 39% (13/33) of the Spanish respondents reported that the impact of the disease on any of the surveyed value dimensions had been evaluated by their HCPs. In contrast, for other countries, more than 79% (United Kingdom: 22/28, 79%, Italy: 24/30, 80%, and Germany: 19/22, 86%) of the respondents reported that at least one of the value dimensions was discussed with their HCP. Also, only 9% to 14% of French (3/23, 13%), Spanish (3/22, 14%), Italian (2/22, 9%), and UK (2/16, 12%) patients had discussed “end-of-life” with their HCPs, while 41% (7/17) of German patients had. Other dimensions also showed variations among countries. French, Spanish, and German patients primarily wanted to evaluate physical functioning and well-being with HCPs, while Italian and UK patients prioritized aspects of emotional well-being (data not shown).

Taken together, the observed differences between gender and countries may serve as examples that indicate considerable differences in value perception between patients or patient subgroups.

### Socioeconomic Impact of LC

The socioeconomic impact of LC was assessed for several aspects of the respondents’ professional lives, household income, and expenditures (Table 4). Most importantly, a substantial economic impact from the disease was reported, despite the existence of comprehensive insurance schemes in all countries involved in the survey.

A majority of patients who were employed at the time of diagnosis saw their employment status impacted by LC. Among patients younger than 65 years, 43% (49/112) had to stop working and 10% (11/112) had to reduce work time because of the disease.

LC also had a negative impact on overall household income. Among the 69 respondents who shared their monthly household income before and after diagnosis, 38 respondents (38/69, 55%) reported a loss of income. Among those 69 respondents, 11 (16%) reported a loss of half or more of their previous household income. Patients also reported that their daily finances were burdened by the costs associated with medical travel (transportation and parking costs, office visits, consultations, and hospitalizations) and costs for alternative medicines and supportive care. Only 25% (37/150) of participants reported having no supplementary costs.

Overall, the daily finances of 69% (103/150) of patients were impacted by LC: 55% (83/150) reported being impacted by a lack of financial assistance or a lack of knowledge about financial assistance; 29% (44/150) of patients reported being impacted due to a lack of support in the procedures to obtain grants or other reimbursements for medical care.

**Table 4:** Economic burden on patients with lung cancer.

			Participants, n (%)
**Impact on employment status (among patients younger than 65 years, n=112)**			
	Unemployed	83 (75)	
	Full-time job	12 (12)	
	Part-time job because of cancer	11 (10)	
	Do not work because of cancer	49 (43)	
	Do not work (not due to cancer)	33 (29)	
	Part-time (not due to cancer)	4 (4)	
	Other	2 (2)	
**Rearrangements of work schedule (among patients working, n=28)**			
	Yes	11 (39)	
	No, I would have liked to	4 (14)	
	No, it has not been necessary	12 (43)	
	I do not know	1 (4)	
**Impact on patients’ monthly household income (among indicated both the household income before and after lung cancer diagnosis, n=69)**			
	1%-70% more	6 (9)	
	No changes	25 (36)	
	1%-25% less	13 (19)	
	26%-50% less	14 (20)	
	51%-100% less	11 (16)	
**Cost covered by patients to manage their condition (n=150)**			
	Transportation costs	61 (41)	
	Alternative medicines	53 (35)	
	Parking costs	52 (35)	
	Supportive care	37 (25)	
	Office visits, consultations, and hospitalizations	36 (24)	
	Home help services	24 (16)	
	Treatment for cancer and side effects	16 (11)	
	Housing adaptation	16 (11)	
	Childcare or care of other dependents	6 (4)	
	Do not know	4 (3)	
	None	37 (25)	
**Monthly household dedicated to managing lung cancer (among patients who answer the complete questions, n=30)**			
	0%-25%	16 (53)	
	26%-50%	5 (17)	
	51%-100%	5 (17)	
	>100%	4 (13)	
**Elements impacting daily finances (n=150**)			
	Lack of financial assistance	58 (39)	
	Lack of knowledge about financial assistance	57 (38)	
	Lack of support for procedures	44 (29)	
	Difficulties to access financial services	27 (18)	
	Complexity of procedures for reimbursement of medical care	21 (14)	
	Increased cost of health insurance	19 (13)	
	Reimbursement delay	13 (9)	
	Other	5 (3)	
	None	47 (31)	

## Discussion

### Principal Findings

To date, most studies assessing QoL in LC care focus on the impact of novel treatments, such as those in clinical trials [6,7,17,21-23,31,32,46]. Therefore, this study adds important insights to the scarce information on QoL priorities of patients with LC in a wider context of care as well as daily life [18,36,47].

All QoL dimensions and subdimensions defined in the study questionnaire received high scores from survey participants. Hence, the set of dimensions explored in the questionnaire appears adequate for capturing topics patients and caregivers consider important. Meanwhile, there were several limitations to the study. Importantly, the study participants were subject to a selection bias, with Carenity members not representing the general population of patients with LC and the study respondents constituting a voluntary sample among Carenity members. The survey respondents were younger than the global population of patients with LC (mean age was 58.8 vs 71 for the global population); a high proportion of patients (81/150, 54%) in our study were also in an early stage of the disease (compared to 15%-30% at diagnosis in the general population [48]), were in remission (40/150, 27%) or were recently diagnosed (90/150, 61% diagnosed within the year). LC is often diagnosed at an advanced stage, where the prognosis is worse. The Carenity members who took part in this survey were also people likely to spontaneously turn to digital media to seek information on their disease, and thus the selected sociocultural population is targeted [49,50]. Furthermore, our study has a limited sample size of 150 participants, with 22 to 37 respondents in each participating country and missing values for some of the variables assessed. However, our main findings were unlikely to be affected by these biases. For example, the importance of various value dimensions was analyzed in relative terms, with end-of-life care consistently showing the highest ratings across respondent groups. End-of-life care was among the dimensions rated the highest for patients in curative settings, for whom intuitively, this aspect of care could be less relevant. Together, this corroborates the high importance of this dimension of care to a majority of patients with LC. Similarly, regarding the socio-economic consequences of LC, the reported data show a substantial negative impact for a considerable proportion of the respondents; this finding remains valid and relevant, even if biased towards specific patient population groups.

In our study, “physical well-being and functioning” was the QoL dimension most frequently discussed with HCPs (82/150, 55%). This topic was also indicated to be the most important dimension to be evaluated by HCPs (114/150, 76%). Only 17% (17/100) of patients reported discussing “end-of-life care” with their HCP, while 45% (45/100) of patients considered it important to do so. The highest overall value score of “end-of-life care” may also reflect the poor prognosis and the high psychological distress incurred by LC, especially in the advanced stages of the disease [51,52]. However, interestingly, this dimension also scored highly for patients with localized disease (median 9.2, IQR 7.7-10.0, data not shown). The high score of “physical well-being and functioning” was unsurprising given the major impact on QoL of the subdimensions “physical well-being,” “mobility,” and “autonomy.” Patient independence has previously been identified as an important determinant of treatment success for LC, while the ability to remain physically functional is frequently indicated as a major QoL priority by patients [19]. Ultimately, the observed lack of communication about end-of-life care could be attributed to emotional barriers between doctors and patients [53], or to a general perception of end-of-life care falling outside the doctor’s primary remit. Nevertheless, our findings suggest that there is a neglected need for discussions on “end-of-life care” between HCPs and patients.

In line with these findings, QoL and patient preferences with regard to treatment priorities have been reported previously to be poorly discussed in routine medical practice [54,55]. But as other studies have shown, the integration of PROMs in clinical routines can improve patient-clinician communication, quality of care, and outcomes [54]. Fortunately, there is a growing interest within the medical community to measure QoL using PROMs and other tools, and some efforts have been made to develop training tools to enhance communication with patients with cancer [12,55]. For example, the US National Cancer Institute sponsors the “Oncotalk” program specifically to provide training in communication skills to oncology physicians [56]. Additionally, the scores for the subdimension “relationship with health care professionals” (9.3, Table 2) were the second highest reported, further pointing to the importance of improved HCP-patient communication.

While patients uniformly indicated a high importance for many QoL dimensions, our findings also illustrated significant differences in value perception among patient groups. For example, women scored higher than men in all QoL dimensions (except “romantic relationship” and “sexual life” subdimensions), and different European countries reported differences in communication practices regarding QoL with HCPs (such as on end-of-life care; Table 2). Technical aspects notwithstanding, differences in value perception may also be exemplified by many PRO instruments not covering or underrepresenting some of the QoL dimensions most highly ranked by patients.

Given their role in medical assessments, these tools may mostly represent clinical priorities, which may not be completely aligned with the priorities of patients and caregivers (Textbox 1). Taken together, our findings indicate a large diversity in value perceptions among various groups of patients, caregivers, and HCPs. This may also highlight the need to assess individual patient perceptions for personalized treatments and further suggest the importance of more broadly assessing the value of LC care beyond purely medical parameters.

Besides the clinical impact of LC, our results indicated a considerable socioeconomic impact of LC for patients and caregivers, despite the coverage of direct costs by insurance schemes in all countries surveyed (49/112, 43% of patients had to stop working due to the cancer and 11/112, 10% of them transitioned to only part-time work). LC also had a negative impact on overall household income, regardless of the patient’s employment status. Among respondents who shared their monthly household income before and after diagnosis, 55% (38/69) of them reported a loss of income. Beyond individual patients, as other reports have cited, the economic burden of cancer on society is substantial, with 60% of this burden incurred in non–health care areas [33].

### Conclusions

This study broadly assessed the importance of different QoL dimensions beyond clinical parameters. End-of-life care was identified to be of highest relevance to patients with LC irrespective of stage at diagnosis; however, this aspect is least frequently discussed with HCPs. The study also indicated a considerable socioeconomic impact of LC despite coverage of direct costs. Moreover, our results suggest an important diversity of value perceptions for different QoL dimensions among groups of patients, caregivers, and HCPs, calling for more personalized treatment decisions and enhanced doctor-patient communication.

## Appendix

Multimedia Appendix 1 Patient recruitment on Carenity.

Multimedia Appendix 2 Survey questionnaire.

Multimedia Appendix 3 Caregivers characteristics.

Multimedia Appendix 4 Comprehensive scoring table by sub-dimensions for the study population.

## Abbreviations

HCP: health care professional
HRQOL: health-related quality of life
ICHOM: International Consortium for Health Outcomes Measurement
LC: lung cancer
PROM: patient-reported outcome measure
QoL: quality of life
