# Supplementary material for: Patient Perspectives on Value Dimensions of Lung Cancer Care: Cross-sectional Web-Based Survey
Source: JMIR Form Res. 2023 Jan 26;7:e37190. doi: 10.2196/37190 (PMC9912155; doi:10.2196/37190)
Supplement: Multimedia Appendix 1 [file formative_v7i1e37190_app1.docx]

**Appendix 1 – Patient recruitment on Carenity**

Carenity is a free, multilingual global online community created in 2011 to help patients and their families share their experiences. Carenity is currently available in six countries (France, the United Kingdom, Italy, Spain, Germany, and the United States). As of June 2022, there are over 500,000 registered members across more than 900 chronic diseases.

Registration on Carenity is free and the platform is accessible to anyone over 18 years of age with an internet connection and an email address. Registration is available on computer, tablet, and mobile devices and is compatible with all operating systems. To register on Carenity, members must provide an email address, a username, and a year of birth, as well as consent to the Terms of Use, formalised by a tick box, and give their explicit consent to the processing of their personal health data, after having read the terms of use via a separate tick box.

When registering on Carenity, patients can give their consent, via an opt-in, to be solicited to participate in online studies related to their condition. Upon registration, new members self-report a chronic condition and their relationship to it (patient, caregiver, or interested party) and join the corresponding community.

To increase recruitment potential, Carenity generally uses both free and paid methods:

- Organic recruitment: Carenity’s website is indexed on search engines (SEO)
- Partnerships and cross-promotion with other health websites (e.g., blogs, forums, Facebook groups, Instagram), can also be implemented with patient organizations.
- Paid recruitment (online campaigns): targeted online advertisements about Carenity

are displayed on Google and Facebook.

Clarifications on Facebook ads and Google ads

**Facebook ads**

As Facebook is a social network present in most countries of the world, it constitutes an

attractive source of recruitment for this project.

Each country has a dedicated campaign containing a link that automatically leads to

the survey. To this end, the first step is to create an audience to target people with the disease of interest. Facebook offers a few powerful methods to target the correct audience for ads:

- Targeting people based on their interests.
- Generating a specific audience using Carenity’s audience of people with an interest in the disease of interest (this method is called “lookalike audience”).

Once the campaign is live, people from the targeted audience see the ad in their Facebook feed. When a Facebook user clicks on the advertisement, he or she is redirected to the survey.

**Google Ads**

Google Ads is an online advertising platform developed by Google, where advertisers bid to display advertisements to web users in the Google Search results pages.

Carenity uses Google Ads to gain visibility and recruit new patients that can integrate

Carenity communities.
