# Supplementary material for: Patient Perspectives on Value Dimensions of Lung Cancer Care: Cross-sectional Web-Based Survey
Source: JMIR Form Res. 2023 Jan 26;7:e37190. doi: 10.2196/37190 (PMC9912155; doi:10.2196/37190)
Supplement: Multimedia Appendix 2 [file formative_v7i1e37190_app2.docx]

**Appendix 2 : Survey questionnaire**

**Defining Value in Health: Evidence generation on cancer – Perspective of Patients and their Caregivers**

**Questions for all - Questions only for patients - Questions only for caregivers**

# Socio-demographic and medical profile

1. **You are:**

*(Please answer only one)*

- Male
- Female

1. **Your year of birth:** (YYYY) *[Calendar choice]* [if <18 years old, end of the survey]
2. **Your country of residence:**

*(Please answer only one)*

- France
- Germany
- Italy
- United Kingdom
- Spain
- Other ***-> Specify:*** *[Free field]* [end of the survey]

1. **You are:**

*(Please answer only one)*

- A patient affected by primary lung cancer (cancer started in the lung)
- A caregiver of a patient affected by primary lung cancer (cancer started in the lung)
- Neither of the above [end of the survey]

[questions q5 to q7 are only for caregiver of a patient affected by lung cancer]

1. **What is your relationship with the patient you provide care for?**

*(Please answer only one)*

- The patient is my partner/spouse
- The patient is my child
- The patient is my mother/my father
- The patient is my brother/sister
- The patient is another member of my family
- The patient is my friend
- I am an employed helper
- Other ***-> Specify:*** *[Free field]*

1. **The patient you provide care for is a:**

*(Please answer only one)*

- Male
- Female

1. **What is the year of birth of the patient you provide care for?**

(YYYY) *[Calendar choice]* [if <18 years old, end of the survey]

1. **What is your highest level of education?**

**What is the highest level of education of the patient you provide care for?**

*(Please answer only one)*

- Did not finish high school
- High school diploma
- 2-year college degree
- Bachelor’s degree
- Master’s degree
- PhD
- Other ***->*** ***Specify:*** *[Free field]*
- I do not know

1. **You are:**

**The patient you provide care for is:**

*(Please answer only one)*

- Single
- In a relationship and living with your/their partner
- In a relationship and not living with your/their partner

1. **You are:**

**The patient you provide care for is:**

*(Please answer only one)*

- Non-smoker
- Former smoker who stopped before the lung cancer diagnosis **->** ***When did you/the patient stop smoking?*** (YYYY) *[Calendar choice]*
- Former smoker who stopped after the lung cancer diagnosis **->** ***When did you/the patient stop smoking?*** (YYYY) *[Calendar choice]*
- Smoker
- I do not know

1. **When was your lung cancer diagnosed?**

**When was the patient diagnosed with lung cancer?**

(MM/YYYY) *[Calendar choice]*

- I do not remember

1. **Do you know the stage of your lung cancer today?**

**Do you know the stage of the patient’s lung cancer today?**

*(Please answer only one)*

- Localised (the cancer is still inside the lung or the cancer has spread into tissues around the lung such as nearby lymph nodes, diaphragm, windpipe or heart tissues)
- Advanced/Metastatic (the cancer is in both lungs and has spread to another part of the body e.g.: liver, brain, bones. The cancer in another part of the body is called a metastasis)
- Other ***->*** ***Specify:*** *[Free field]*
- I do not know
- I do not want to answer

1. **Do you know if your lung cancer is…?**

**Do you know if the patient’s lung cancer is…?**

*(Please answer only one)*

- Active under curative care
- Active under palliative care
- In remission
- I do not know
- I do not want to answer

1. **Which treatments are you currently receiving for your lung cancer?**

**Which treatments is the patient currently receiving for their lung cancer?**

*(Select all that apply)*

- Surgery (during the last month)
- Radiotherapy (type of cancer treatment that uses a high dose of radiation to kill cancer cells and shrink tumours)
- Chemotherapy (type of cancer treatment that uses drugs to kill cancer cells)
- Immunotherapy (type of cancer treatment that helps the immune system fight cancer)
- Targeted therapy (type of cancer treatment that targets the changes in cancer cells that help them grow, divide and spread)
- I do not know
- I am not being treated for my lung cancer
- The patient is not being treated for their lung cancer

1. **Have you ever participated in a clinical trial for your lung cancer**?

**Has the patient ever participated in a clinical trial for their lung cancer?**

*(Please answer only one)*

*Clinical trials are medical research studies that involve people. New drugs are first studied in a laboratory, and those that look promising go on to be carefully studied in people through clinical trials. The aim of a clinical trial is to test new drugs to see how well they work in helping people feel better, or in helping a disease to improve or stop getting worse.*

- Yes, currently
- Yes, in the past
- No, never
- I do not know

1. **In relation to your disease, to what extent are the following aspects of your physical functioning and well-being of major importance to you?**

*Please drag the slider to the desired position (left end = not important, right end = very important)*

**In relation to their disease, to what extent are the following aspects of the patient’s physical functioning and well-being of major importance to them?**

*Please drag the slider to the desired position (left end = not important, right end = very important)*

*(Only 1 answer per line – Randomized question)*

| Physical well-being (symptoms, side effects…) | not important ----------------O----------------- very important |
| --- | --- |
| Autonomy (dress, wash yourself, cook…) | not important ----------------O----------------- very important |
| Mobility (physical limitations, ability to walk, drive, take transportation…) | not important ----------------O----------------- very important |

1. **Has the impact of lung cancer on your physical functioning and well-being ever been evaluated by healthcare professionals?**

**Has the impact of lung cancer on the patient’s physical functioning and well-being ever been evaluated by healthcare professionals?**

*(Please answer only one)*

- Yes
- No
- I do not know

1. **In relation to your disease, to what extent are the following aspects of your emotional well-being of major importance to you?**

*Please drag the slider to the desired position (left end = not important, right end = very important)*

**In relation to their disease, to what extent are the following aspects of the patient’s emotional well-being of major importance to them?**

*Please drag the slider to the desired position (left end = not important, right end = very important)*

*(Only 1 answer per line – Randomized question)*

| Emotional well-being (self-esteem, mood, emotional state…) | not important ----------------O----------------- very important |
| --- | --- |
| Emotional support from family and friends | not important ----------------O----------------- very important |
| Self-acceptance (not feeling guilty) | not important ----------------O----------------- very important |
| Not being judged/blamed by others | not important ----------------O----------------- very important |

1. **Has the impact of lung cancer on your emotional well-being ever been evaluated by healthcare professionals?**

**Has the impact of lung cancer on the patient’s emotional well-being ever been evaluated by healthcare professionals?**

*(Please answer only one)*

- Yes
- No
- I do not know

1. **In relation to your disease, to what extent are the following aspects of your daily life of major importance to you?**

*Please drag the slider to the desired position (left end = not important, right end = very important)*

**In relation to their disease, to what extent are the following aspects of the patient’s daily life of major importance to them?**

*Please drag the slider to the desired position (left end = not important, right end = very important)*

*(Only 1 answer per line – Randomized items)*

| Social life (frequency of outings, social network…) | not important ----------------O----------------- very important |
| --- | --- |
| Family life (quality of family time, holidays…) | not important ----------------O----------------- very important |
| Romantic relationship | not important ----------------O----------------- very important |
| Sexual life | not important ----------------O----------------- very important |
| Leisure (sport, cinema, outings…) | not important ----------------O----------------- very important |
| Purchasing power/ standard of living | not important ----------------O----------------- very important |
| Professional life (scheduling, promotions, work interruptions…) | not important ----------------O----------------- very important |

1. **Has the impact of lung cancer on your daily life ever been evaluated by healthcare professionals?**

**Has the impact of lung cancer on the patient’s daily life ever been evaluated by healthcare professionals?** *(Please answer only one)*

- Yes
- No
- I do not know

1. **In relation to your disease, to what extent are the following aspects of your medical care of major importance to you?**

*Please drag the slider to the desired position (left end = not important, right end = very important)*

**In relation to their disease, to what extent are the following aspects of the patient’s medical care of major importance to them?**

*Please drag the slider to the desired position (left end = not important, right end = very important)*

| Easy access to place of care (travel time, transportation…) | not important ----------------O----------------- very important |
| --- | --- |
| Less overnight time spent at place of care (number of hospitalisations and duration) | not important ----------------O----------------- very important |
| Low frequency of medical follow-up (consultation, blood tests, examinations...) | not important ----------------O----------------- very important |
| Relationship with healthcare professionals (communication, empathy…) | not important ----------------O----------------- very important |

*(Only 1 answer per line – Randomized question)*

1. **Has the impact of your medical care on your quality of life ever been evaluated by healthcare professionals?**

**Has the impact of the patient’s medical care on their quality of life ever been evaluated by healthcare professionals?**

*(Please answer only one)*

- Yes
- No
- I do not know

1. **In relation to your disease, to what extent are the following aspects of the treatment of major importance to you?**

*Please drag the slider to the desired position (left end = not important, right end = very important)*

**In relation to their disease, to what extent are the following aspects of the patient’s treatment of major importance to them?**

*Please drag the slider to the desired position (left end = not important, right end = very important)*

| Possibility to take the treatment by myself/ by themselves | not important ----------------O----------------- very important |
| --- | --- |
| Convenience of the route of administration | not important ----------------O----------------- very important |
| Treatment side effects | not important ----------------O----------------- very important |
| Logistics to get the treatment | not important ----------------O----------------- very important |

*(Only 1 answer per line – Randomized question)*

1. **Has the impact of your treatment on your quality of life ever been evaluated by healthcare professionals?**

**Has the impact of the patient’s treatment on their quality of life ever been evaluated by healthcare professionals?**

*(Please answer only one)*

- Yes
- No
- I do not know

1. **With respect to the life of your caregivers, to what extent are the following aspects of their daily life of major importance to them?**

*Please drag the slider to the desired position (left end = not important, right end = very important)*

*(Only 1 answer per line – Randomized question)*

| Caregiver’s emotional well-being (mood, emotional state…) | not important ----------------O----------------- very important |
| --- | --- |
| Caregiver’s physical well-being (physical condition, fatigue…) | not important ----------------O----------------- very important |
| Caregiver’s family life (quality of family time, holidays…) | not important ----------------O----------------- very important |
| Caregiver’s romantic relationship/private life | not important ----------------O----------------- very important |
| Caregiver’s purchasing power/standard of living | not important ----------------O----------------- very important |
| Caregiver’s professional life (scheduling, promotions, work interruptions…) | not important ----------------O----------------- very important |

**26bis. With respect to your life as a caregiver, to what extent are the following aspects of your daily life of major importance to you?**

*Please drag the slider to the desired position (left end = not important, right end = very important)*

*(Only 1 answer per line – Randomized question)*

| Your emotional well-being (mood, emotional state…) | not important ----------------O----------------- very important |
| --- | --- |
| Your physical well-being (physical condition, fatigue…) | not important ----------------O----------------- very important |
| Your family life (quality of family time, holidays…) | not important ----------------O----------------- very important |
| Your romantic relationship/private life | not important ----------------O----------------- very important |
| Your professional life (scheduling, promotions, work interruptions…) | not important ----------------O----------------- very important |
| Your purchasing power/standard of living | not important ----------------O----------------- very important |

1. **Has the impact of lung cancer on your caregiver’s daily life ever been evaluated by healthcare professionals?**

**Has the impact of lung cancer on your daily life as a caregiver ever been evaluated by healthcare professionals?**

*(Please answer only one)*

- Yes
- No
- I do not know

1. **Are you willing to answer a set of questions on the subject of end-of-life care?**

**Have you ever discussed with the patient on the subject of end-of-life care and are you willing to answer a set of questions on this subject?**

*(Please answer only one)*

- Yes
- No -> Q31

[Q29 will be a non-compulsory question]

1. **In relation to your disease, to what extent are the following aspects of end-of-life care of major importance to you?**

*Please drag the slider to the desired position (left end = not important, right end = very important)*

*This question is not mandatory. You may decide not to answer some aspects or not to answer the question at all.*

**In your opinion, in relation to the patient’s disease, to what extent are the following aspects of end-of-life care of major importance to them?**

*Please drag the slider to the desired position (left end = not important, right end = very important)*

*This question is not mandatory. You may decide not to answer some aspects or not to answer the question at all.*

| Place of death (home, hospital…) | not important ----------------O----------------- very important |
| --- | --- |
| Presence of loved ones at moment of death | not important ----------------O----------------- very important |
| Pain management | not important ----------------O----------------- very important |
| End-of life assistance (psychological support, administrative, financial…) | not important ----------------O----------------- very important |
| Duration of end-of-life hospitalisation | not important ----------------O----------------- very important |
| Involvement in end-of-life care decisions/respect of living will | not important ----------------O----------------- very important |
| Financial impact on loved ones | not important ----------------O----------------- very important |

*(Only 1 answer per line – Randomized question)*

1. **Have you ever discussed end-of-life care with healthcare professionals?**

**Has the patient ever discussed end-of-life care with healthcare professionals?**

*(Please answer only one)*

- Yes
- No
- I do not know

**[Q31 is only for patients who discussed/evaluated at least one of the previous aspects with healthcare professionals]**

1. **When did healthcare professionals evaluate the impact of lung cancer on your quality of life?**

*(Examples: impact on* *physical functioning and well-being, emotional well-being, daily life, impact of medical care and treatments on your quality of life, impact on caregiver’s daily life, end-of-life care…)*

**When did healthcare professionals evaluate the impact of lung cancer on the patient’s quality of life?**

*(Examples: impact on* *physical functioning and well-being, emotional well-being, the patient’s daily life, impact of medical care and treatments on the patient’s quality of life, impact on your daily life, end-of-life care…)*

*(Select all that apply)*

- When starting/changing treatment
- When experiencing side effects
- During a hospitalisation
- During a medical check-up
- In the context of a patient support program (therapeutic education program…)
- During a clinical trial
- At another time ***-> Specify:*** *[Free field]*
- I do not know

1. **Which aspects of your life impacted by lung cancer are of major importance to you to evaluate with healthcare professionals?**

**Which aspects of the patient’s life impacted by lung cancer are of major importance to them to evaluate with healthcare professionals?**

*If you select “Other”, please let us know about impacts of lung cancer that are not listed and do not mention any brand or specific treatment.*

*(Select all that apply)*

- Physical functioning and well-being (autonomy, mobility…)
- Emotional well-being (self-esteem, mood, emotional state…)
- Daily life (social life, family life, leisure…)
- Medical care (treatment side effects, care pathway organization…)
- Caregiver’s daily life (emotional state, fatigue…)
- End-of-life care (pain management, end-of-life assistance…)
- Other ***-> Specify:*** *[Free field]*
- I do not know
- None

# Economic burden of lung cancer

1. **Please tell us about your employment status:**

*(Please answer only one)*

- I work full-time
- I work part-time because of my lung cancer
- I work part-time but it is not due to my lung cancer
- I do not work because of my lung cancer
- I do not work but it is not due to my lung cancer (retired, homemaker…)
- Other ***-> Specify:*** *[Free field]*

**33bis. Please tell us about the patient’s employment status:**

*(Please answer only one)*

- The patient works full-time
- The patient works part-time because of their lung cancer
- The patient works part-time but it is not due to their lung cancer
- The patient does not work because of their lung cancer
- The patient does not work but it is not due to their lung cancer (retired, homemaker…)
- Other ***-> Specify:*** *[Free field]*

**[Question 34 is only for patients working full-time or part-time.]**

1. **Have you had to rearrange your working schedule because of your lung cancer?**

**Has the patient had to rearrange their working schedule because of their lung cancer?**

- Yes ***-> Please specify how you/the patient rearranged your/their schedule:*** *[Free field]*
- No, but I/the patient would have liked to
- No, it has not been necessary
- I do not know

1. **What is the average level of monthly income in your household after taxes, including the aid you may receive?**

**What is the average level of monthly income in the patient’s household after taxes, including the aid the patient may receive?**

*This question is only asked to assess loss of income due to lung cancer. You can give us your best approximation or decide not to answer.*

*(Minimum=0)*

Before lung cancer diagnosis: Numeric field, in the local currency

- I do not know or I prefer not to answer

Currently: Numeric field, in the local currency

- I do not know or I prefer not to answer

1. **Which of the following costs (costs not reimbursed by insurance or public provision) do you have to cover yourself to manage your lung cancer?**

**Which of the following costs (costs not reimbursed by insurance or public provision) does the patient have to cover themselves to manage their lung cancer?**

*(Select all that apply – Randomized question)*

- Transportation costs
- Parking costs
- Office visits/consultations/hospitalisations
- Treatment for cancer and for any related side effects
- Supportive care (psychological support, sport, nutrition…)
- Alternative medicines (acupuncture, hypnosis, herbal medicines…)
- Home help services (home nurse, home helper…)
- Childcare or care of other dependants
- Housing adaptation (mobility aids, equipment…)
- I do not know -> Q38
- None -> Q38

1. **Please indicate what you estimate is your total financial cost to manage your lung cancer (without the reimbursements) monthly:**

**Please indicate what you estimate is the patient’s total financial cost to manage their lung cancer (without the reimbursements) monthly:**

*(Minimum=0)*

Numeric field, in the local currency

- I do not know

1. **Which of the following elements impact your daily finances?**

**Which of the following elements impact the patient’s daily finances?**

*(Select all that apply – Randomized question)*

- Reimbursement delay for advanced expenses related to lung cancer
- Lack of knowledge about financial assistance and procedures for patients with lung cancer
- Lack of financial assistance for patients with lung cancer
- Complexity of procedures for grants or reimbursement of medical care
- Lack of support for procedures (social assistance…)
- Difficulties to access financial services (credit, insurance…)
- Increased cost of health insurance due to lung cancer
- I do not know
- Non
