# Supplementary material for: Patient Perspectives on Value Dimensions of Lung Cancer Care: Cross-sectional Web-Based Survey
Source: JMIR Form Res. 2023 Jan 26;7:e37190. doi: 10.2196/37190 (PMC9912155; doi:10.2196/37190)
Supplement: Multimedia Appendix 3 [file formative_v7i1e37190_app3.docx]

**Appendix 3 – Caregivers socio demographic characteristics**

| **Characteristics** | | Caregivers, n. (%)  (N=35) |
| --- | --- | --- |
| **Gender** | |  |
|  | Female | 33 (94%) |
| **Age** | |  |
|  | <46 | 17 (49%) |
|  | 46-55 | 12 (34%) |
|  | 56-65 | 6 (17%) |
| **Relationship with the patient** | |  |
|  | Child of the patient | 16 (45%) |
|  | Partner of the patient | 14 (40%) |
|  | Other family member | 2 (6%) |
|  | Friend | 2 (6%) |
|  | Employed helper | 1 (3%) |
| **Country** | |  |
|  | France | 15 (43%) |
|  | Spain | 11 (31%) |
|  | Italy | 4 (11%) |
|  | UK | 3 (9%) |
|  | Germany | 2 (6%) |
