# Supplementary material for: Patient Perspectives on Value Dimensions of Lung Cancer Care: Cross-sectional Web-Based Survey
Source: JMIR Form Res. 2023 Jan 26;7:e37190. doi: 10.2196/37190 (PMC9912155; doi:10.2196/37190)
Supplement: Multimedia Appendix 4 [file formative_v7i1e37190_app4.docx]

**Appendix 4- Comprehensive scoring table by sub-dimensions for the study population.**

| **QoL** | | **All respondents** | | |  |
| --- | --- | --- | --- | --- | --- |
|  |  |  |  |  |  |
| **Dimension** | **Sub-dimension** | **Number of respondents** | **Median score (Q1-Q3)** | **Mean score (SD)** |  |
| Physical functioning and well-being | | 150 | 9,6(7,7-10) | 8,5(2,2) |  |
|  | Physical well-being |  | 9,4(7,6-10) | 8,5(2,1) |  |
|  | Autonomy |  | 9,8(7,6-10) | 8,4(2,4) |  |
|  | Mobility |  | 9,6(8-10) | 8,6(2,2) |  |
| Emotional well-being |  | 150 | 8,6(5,8-10) | 7,4(3,1) |  |
|  | Emotional well-being |  | 9,3(7,4-10) | 8,3(2,3) |  |
|  | Emotional support from family and friends |  | 9,5(7,6-10) | 8,3(2,6) |  |
|  | Self-acceptance |  | 8,5(5,9-9,9) | 7,3(3,1) |  |
|  | Not being judged/blamed by others |  | 6,6(2,1-9,5) | 5,9(3,7) |  |
| Daily life |  | 150 | 6,9(3,1-9,5) | 6(3,5) |  |
|  | Social life |  | 6,9(4,9-8,7) | 6,3(3) |  |
|  | Family life |  | 8,8(7-10) | 7,9(2,8) |  |
|  | Romantic relationship |  | 6,9(1,6-9,8) | 5,8(3,8) |  |
|  | Sexual life |  | 4,2(1-8) | 4,4(3,5) |  |
|  | Leisure |  | 6,6(4,5-8,7) | 6,1(3,1) |  |
|  | Purchasing power |  | 7,5(5,3-10) | 6,9(3) |  |
|  | Professional life |  | 4,5(0,9-8,3) | 4,7(3,7) |  |
| Medical care |  | 150 | 8,9(6,7-10) | 7,9(2,6) |  |
|  | Easy access to place of care |  | 9,1(7,1-10) | 8,2(2,4) |  |
|  | Less overnight time spent at place of care |  | 8,4(5,2-10) | 7,2(3,1) |  |
|  | Low frequency of medical follow-up |  | 8,5(5,7-10) | 7,7(2,5) |  |
|  | Relationship with healthcare professionals |  | 9,8(8,2-10) | 8,7(2) |  |
| Treatment | | 150 | 8,8(6,4-10) | 7,7(2,8) |  |
|  | Possibility to take the treatment by myself/ by themselves |  | 8,4(4,8-10) | 6,9(3,3) |  |
|  | Convenience of the route of administration |  | 8,7(6,6-10) | 7,8(2,7) |  |
|  | Treatment side effects |  | 9,2(7,7-10) | 8,3(2,3) |  |
|  | Logistics to get the treatment |  | 8,7(6,3-10) | 7,7(2,6) |  |
| End-of-life | | 100 (67%) | 9,7(8,0-10) | 8,1(2,9) |  |
|  | Place of death |  | 9,4(6,4-10) | 7,7(3,3) |  |
|  | Presence of loved ones at moment of death |  | 9,8(8,0-10) | 8,2(3) |  |
|  | Pain management |  | 10(9,2-10) | 9,2(1,9) |  |
|  | End-of life assistance |  | 9,1(6,7-10) | 7,8(3) |  |
|  | Duration of end-of-life hospitalisation |  | 9,5(7,8-10) | 8,1(2,9) |  |
|  | Involvement in end-of-life care decisions/respect of living will |  | 9,7(7,8-10) | 8,3(2,6) |  |
|  | Financial impact on loved ones |  | 9,4(5,6-10) | 7,7(3,2) |  |
